# A Case of Protruding Eyeballs and Diminishing Vision

**DOI:** 10.4137/ccrep.s3752

**Published:** 2010-01-20

**Authors:** Rakesh Biswas, Saroj Gupta, Sumit Dahiya, Shashi Kiran, B.V. Girish, A.S. Kasthuri

**Affiliations:** 1 People’s College of Medical Sciences, Bhopal, India.; 2 Melaka Manipal Medical College, Melaka, Malaysia.; 3 Vydehi Institute of Medical Education and Research, Bangalore, India. Email: rakesh7biswas@gmail.com

**Keywords:** thyroid ophthalmopathy, hypothyroidism, vision loss

## Abstract

This case report discusses issues related to a 56-year-old man from Bangalore who presented with complaints of a gradual protrusion of his eyeballs along with diminishing vision for the previous month. The approach to diagnosis and management issues around this unusual presentation is dicussed.

A 56-year-old man from Bangalore presented with complaints of a gradual protrusion of his eyeballs along with diminishing vision for the previous month. On examination he had proptosis (bilateral-left more than right-Fig. 1) along with bilateral restricted lateral and medial gaze. His right was pseudophakic (lens implantation done) whereas the left eye showed senile cataract. His visual acuity was reduced to finger counting only up to one foot in both eyes. There was no significant peri-orbital edema, scleral injection, or conjunctival chemosis, neither did the patient complain of diplopia. The fundus examination showed significant optic disc pallor in both eyes.

He had bradycardia with pulse rate 56 per minute at rest and a doubtful delayed relaxation of his ankle jerk. His serum free T3, T4 were reduced along with an elevated TSH (twice normal). A detailed ophthalmologic evaluation further revealed raised intraocular tension in both eyes (right 43 mm and left 34 mm Hg). A CT orbit showed swollen extraocular muscles (Figs. 2, 3).

## Learning Points

1. How common is hypothyroidism in Graves ophthalmopathy?
2. What is the chronology of appearance of thyroid involvement in Graves ophthalmopathy?
3. What are the mechanisms of visual loss in severe Graves ophthalmopathy?
  1. **Graves**’ ophthalmopathy is also called *thyroid-associated ophthalmopathy*, as it occurs in the absence of **Graves**’ disease in 10% of patients. Some of these individuals may have hypothyroidism. In a recent retrospective study of 482 patients, 413 were classified as having ophthalmopathy associated with some form of thyroid dysfunction (dysthyroid group) and of these 413 patients, 401 had a documented history of **Graves’** hyperthyroidism. Hashimoto’s was previously diagnosed in four in which two also had Graves’ disease. Eight had a history of hypothyroidism of unknown aetiology. The remaining 69 patients were found to be euthyroid.^1^
  2. The onset of **Graves**’ ophthalmopathy occurs within the year before or after the diagnosis of thyrotoxicosis in 75% of patients but can sometimes precede or follow thyrotoxicosis by several years, accounting for some cases of euthyroid ophthalmopathy.
    Many patients with **Graves**’ disease have little clinical evidence of ophthalmopathy although enlarged extraocular muscles typical of the disease, and other subtle features, can be detected in almost all patients when investigated by ultrasound or computed tomography (CT) imaging of the orbits. Unilateral signs are found in up to 10% of patients.^2^
    In a recent study of chronology of graves ophthalmopathy among 108 patients with hyperthyroidism, ophthalmopathy was diagnosed in the six-month interval preceding the diagnosis of thyroid dysfunction in 20 patients (18.5%); ophthalmopathy was concurrent with the diagnosis of hyperthyroidism in 22 patients (20.3%); and ophthalmopathy developed in the six-month interval after thyroid diagnosis in 24 patients (22.2%). Ophthalmopathy was diagnosed more than six months before the diagnosis of hyperthyroidism in only four additional patients (3.7%), whereas ocular changes developed six months or more after thyroid disease in the remaining 38 patients (35.2%).^2^
  3. The clinical course of ophthalmopathy does not follow that of the thyroid disease. Ophthalmopathy typically worsens over the initial 3 to 6 months, followed by a plateau phase over the next 12 to 18 months, with spontaneous improvement, particularly in the soft tissue changes. The course is more severe in up to 5% of patients, requiring intervention in the acute phase if there is optic nerve compression or corneal ulceration. Extreme forms of ophthalmopathy, can threaten vision. The forms include subluxation of the globe due to severe proptosis, ulceration or infection of the cornea secondary to an inability to close the lids, and optic neuropathy caused by compression of the optic nerve at the apex of the orbit. Orbital congestion associated with thyroid orbitopathy may produce an increase in IOP by elevation of episcleral venous pressure (EVP) and may benefit with early decompression surgery.^3^ A statistically significant association between hypothyroidism and primary open-angle glaucoma has been demonstrated in prospective case control studies.^4^ Our index case demonstrated evidence of glaucoma possibly related to swollen extra ocular muscles and other intra orbital contents due to immune mediated inflammation. He was unable to afford further investigations. He had substantial visual loss already on presentation and was put on high dose steroids after which the intraocular tension came down in 3 days to normal levels although there was no improvement of visual acuity. He had presented late, had already developed significant optic atrophy secondary to compressive optic neuropathy and secondary glaucoma. As even high dose of steroids did not show any improvement in visual acuity, orbital decompression was not planned.

## Figures and Tables

**Figure 1:**
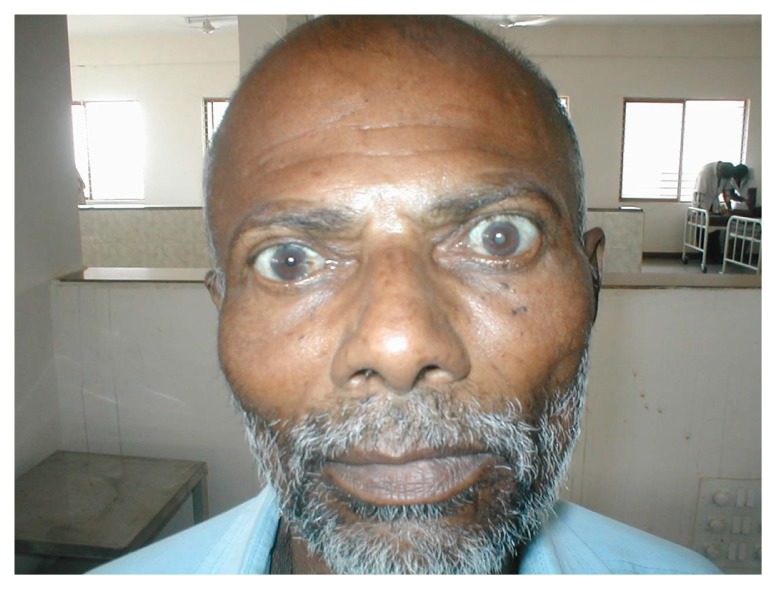
Proptosis: (bilateral-left more than right) with lens implantation in right eye and cataract in left eye.

**Figure 2:**
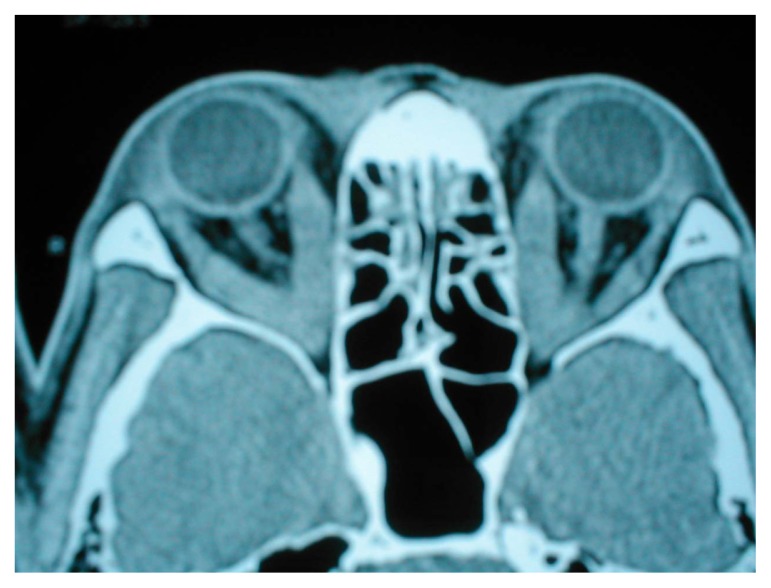
CT orbit showing swollen extra-ocular muscles in both eyes.

**Figure 3:**
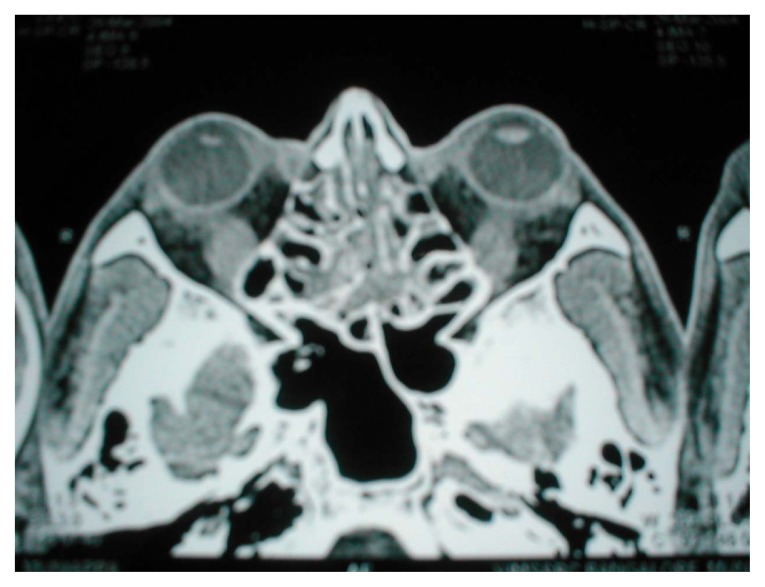
CT orbit showing swollen inferior rectus muscle in both eyes.
